# Is an individual journal article a tiger or a fox?

**DOI:** 10.1186/s40902-018-0186-9

**Published:** 2019-01-23

**Authors:** Seong-Gon Kim

**Affiliations:** Department of Oral and Maxillofacial Surgery, College of Dentistry, Gangneung-Wonju National University, Gangneung, 28644 Republic of Korea

In Asian folklore, there is a story about a fox and a tiger. The fox told the tiger that the fox was a king. To prove this, the fox said he would stroll in the forest and the tiger must stay behind him. Many animals ran away when they saw the tiger behind the fox. The tiger thought they ran because of the fox. This story indicates that the fox behaved like the tiger by borrowing the tiger’s identity. This is analogous to the publication of scientific articles because of errant governmental policies.

Some authors strive to publish their articles in high-impact journals for promotional reasons. Even when this is successful, not every article in high-impact journals has the same impact on the journal [1, 2]. The impact factor was originally developed to evaluate journals, not individual articles. A low percentage of articles has a significant effect on the journals’ performance. Major journals are also dependent on the influence of notable articles. If the top and bottom 5% of articles are excluded from impact factor calculations, the gap among journals in the same field narrows significantly. Thus, many countries evaluate articles without considering the journal impact factor.

The journal impact factor is calculated by the number of citations in the Web of Science; this is not the total number of citations but mutual citations among the indexed journals. This is the same for Scopus. Accordingly, the impact factor is inaccurate and considerably different than cross-reference-based calculations. This is particularly high in regional journals. There are also many flaws associated with citation data [2]. The development of fair evaluation systems by non-profit organizations for journals and articles should be encouraged. The Korean Citation Index (KCI) may be a fair evaluation system, but it has significant limitations similar to the Web of Science and Scopus because it calculates the impact factor among KCI-indexed journals.

Much research funding is provided by the Korean government, which evaluates the success of a project by counting the number of published articles in high-impact journals. Similar evaluations also occur in China. This policy boosts impact factor inflation or potential manipulation. Therefore, government policies should be changed. New evaluation tools for individual articles should be developed and implemented. The journal impact factor should be used to evaluate journals, not individual articles.
